# Peer Review of “Predicting Waist Circumference From a Single Computed Tomography Image Using a Mobile App (Measure It): Development and Evaluation Study”

**DOI:** 10.2196/54011

**Published:** 2023-12-12

**Authors:** Zied Hadrich

**Affiliations:** 1 Department of Surgery, Faculty of Medicine of Tunis, Tunis, Tunisia

**Keywords:** waist circumference, computed tomography, abdominal CT, mobile health, health apps, CT, CT scan, CT image, mobile app, app, application, waist, body, body mass, BMI, morbidity, mortality, clinical, tool, prototype, design, obesity, abdominal, usability, validity, medical

*This is a peer-review report submitted for the paper “Predicting Waist Circumference From a Single Computed Tomography Image Using a Mobile App (Measure It): Development and Evaluation Study.”*

## Round 1 Review

This manuscript [1] is well written.

This paper presents an original idea to simplify patient care. It can be generalized to other specialties.

No specific comments.

### Major Comment

This mobile app could be used for other measurements.
